# Blood cell parameters and risk of nonalcoholic fatty liver disease: a comprehensive Mendelian randomization study

**DOI:** 10.1186/s12920-024-01879-7

**Published:** 2024-04-23

**Authors:** Nan Zhu, Xiaoliang Wang, Huiting Zhu, Yue Zheng

**Affiliations:** 1 Department of Internal Medicine, Hebei Medical University, 050017 Shijiazhuang, Hebei Province China; 2 Department of Internal Medicine, The First Hospital of Qinhuangdao, 066000 Qinhuangdao, Hebei Province China; 3 Department of Cardiology, The First Hospital of Qinhuangdao, 066000 Qinhuangdao, Hebei Province China; 4 Department of Gastroenterology, The First Hospital of Qinhuangdao, 066000 Qinhuangdao, Hebei Province China

**Keywords:** Blood cell indicators, NAFLD, Causality, NHANES, Inflammation

## Abstract

**Background:**

Nonalcoholic fatty liver disease (NAFLD) is on the rise globally, and past research suggests a significant association with various blood cell components. Our goal is to explore the potential correlation between whole blood cell indices and NAFLD risk using Mendelian randomization (MR).

**Methods:**

We analyzed data from 4,198 participants in the 2017–2018 National Health and Nutrition Examination Survey to investigate the link between blood cell indicators and NAFLD. Using various methods like weighted quantile sum and multivariate logistic regression, we assessed the association. Additionally, two-sample Mendelian randomization were employed to infer causality for 36 blood cell indicators and NAFLD.

**Results:**

Multivariate logistic regression identified 10 NAFLD risk factors. Weighted quantile sum revealed a positive correlation (*p* = 6.03e-07) between total blood cell indices and NAFLD, with hemoglobin and lymphocyte counts as key contributors. Restricted cubic spline analysis found five indicators with significant nonlinear correlations to NAFLD. Mendelian randomization showed a notable association between reticulocyte counts and NAFLD using the inverse-variance weighted method.

**Conclusions:**

Hematological markers pose an independent NAFLD risk, with a positive causal link found for reticulocyte count. These results emphasize the importance of monitoring NAFLD and investigating specific underlying mechanisms further.

**Supplementary Information:**

The online version contains supplementary material available at 10.1186/s12920-024-01879-7.

## Introduction

Nonalcoholic fatty liver disease (NAFLD) is the hepatic manifestation of metabolic dysfunction, encompassing liver pathologies like simple steatosis, steatohepatitis, fibrosis, and cirrhosis [1, 2]. NAFLD had a global prevalence of 25.3% in 1990–2006 to 38.0% in 2016–2019, and is expected to increase considerably with the growing and aging population [3]. Therefore, it is crucial to increase NAFLD surveillance to avoid societal burdens associated with the disease and its progression [4]. However, NAFLD has a complex etiology involving many risk factors and genetic susceptibilities that are not fully understood [5]. An urgent need exists for a deeper understanding of the causality and varying effect sizes of different NAFLD risk factors. NAFLD onset and progression are linked to metabolic, inflammatory, genetic, and environmental factors, resulting in prolonged immune system activation and mild inflammation, contributing to chronic organ inflammation strongly correlated with NAFLD [6]. Recently, the nomenclature of NAFLD has been updated to metabolic dysfunction-associated steatotic liver disease (MASLD), which includes the presence of at least 1 of 5 cardiometabolic risk factors, to provide a more comprehensive definition and better understanding of its pathogenesis [7]. 

Blood components and associated cytokines play vital roles in the diagnosis and progression of metabolic diseases. While previous studies have identified correlations between specific blood cell components and NAFLD, the overall involvement of blood cells in NAFLD needs further elucidation [8–10]. No clear correlation exists between overall blood cells and NAFLD development. The role of specific blood cell markers in NAFLD pathogenesis remains unclear. Importantly, a close connection exists between circulating inflammatory factors and blood cells [11]. Studies have highlighted the significant presence of inflammatory factors in NAFLD [8, 11]. However, large-sample, multidimensional studies are needed to establish a causal link among these components.

National Health and Nutrition Examination Survey (NHANES) evaluates the health and nutrition status in the USA, widely used in observational studies. These studies face challenges like bias and confounding. Mendelian randomization (MR) simulates a randomized controlled trial, minimizing biases by leveraging random gene assignment unaffected by population or environment [12]. Combining MR with NHANES enhances research reliability, allowing precise evaluation of exposure impact on outcomes while considering confounders. This contributes to improved public health and quality of life.

This study investigated the correlation between blood cell indices and NAFLD using two methods: observational analysis with NHANES data and Mendelian randomization. Multivariate logistic regression and weighted quantile sum assessed their influence, while a restricted cubic spline model examined potential nonlinear relationships. Despite observational limitations, MR provided comprehensive evidence for a potential causal link between blood cell indicators and NAFLD.

## Methods

### Study population

**Fig. 1:**
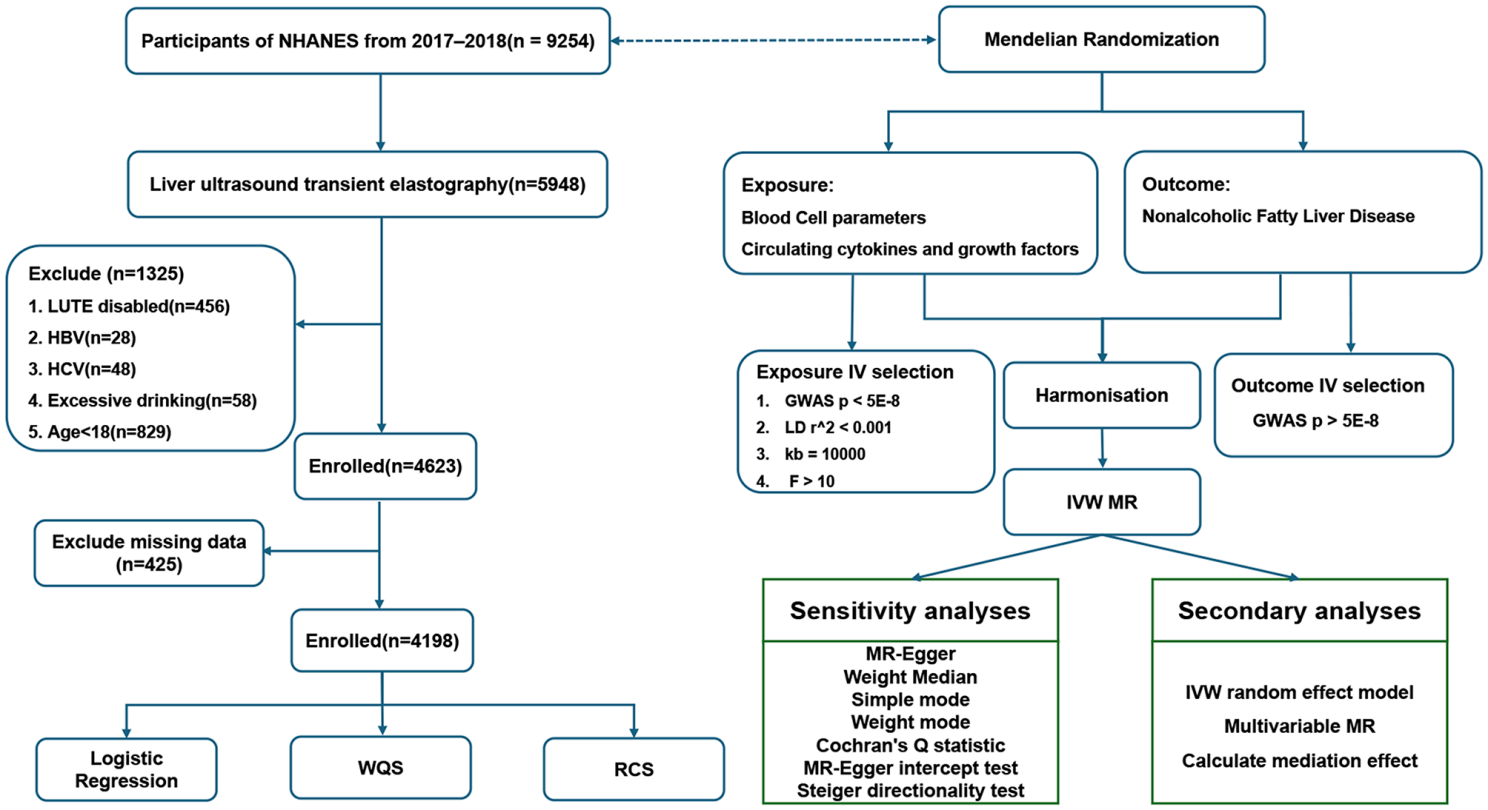
Overall flow chart. NHANES: National Health and Nutrition Examination Survey; BMI: Body Mass Index; WQS: Weighted Quantile Sum; RCS: Restricted cubic spline; MR: Mendelian Randomization; IVW: Inverse-variance weighted

We utilized NHANES data from the National Center for Health Statistics, previously published at https://wwwn.cdc.gov/Nchs/Nhanes/ [13, 14]. We analyzed data from 9254 NHANES 2017–2018 subjects, focusing on 5948 participants with liver steatosis identified through ultrasound transient elastography. Fatty liver was indicated by a median CAP value above 274 dB/m (sensitivity 90%, specificity 60%) [15]. NAFLD was diagnosed with a CAP > 274 dB/m, excluding those with incomplete elastography (*n* = 456), positive for hepatitis B or C (*n* = 76), and heavy alcohol consumption (*n* = 58; women > 2 drinks/day, men > 3 drinks/day) [16]. Excluding participants under 18 years (*n* = 829), 4623 subjects underwent follow-up analysis. After removing 425 cases with missing blood cell exams and covariates (education, BMI, hypertension, and diabetes), the final study cohort comprised 4198 cases, as illustrated in Fig. 1.

### Blood cell-related indicators and covariates

The study included 20 blood cell-related indicators: LBXWBCSI-White blood cell count (1000 cells/µL), LBXLYPCT-Lymphocyte percent (%), LBXWBCSI-White blood cell count (1000 cells/µL), and LBXLYPCT-lymphocyte percent (%). LBXMOPCT-Monocyte percent (%), LBXNEPCT-Segmented neutrophils percent (%), LBXEOPCT-Eosinophils percent (%), LBXBAPCT-Basophils percent (%), LBDLYMNO-Lymphocyte number (1000 cells/µL), LBDMONO-Monocyte number (1000 cells/µL), LBDNENO-Segmented neutrophils num (1000 cells/µL), LBDEONO-Eosinophils number (1000 cells/µL), LBDBANO-Basophils number (1000 cells/µL), LBXRBCSI-Red blood cell count (million cells/µL), LBXHGB-Hemoglobin (g/dL), LBXHCT-Hematocrit (%), LBXMCVSI-Mean cell volume (fL), LBXMCHSI-Mean cell hemoglobin (pg), LBXMC-MCHC (g/dL), LBXRDW-Red cell distribution width (%), LBXPLTSI - Platelet count SI (1000 cells/µL), and LBXMPSI - Mean platelet volume (fL). Ten covariates were assessed as potential confounders: sex, age, race, poverty–income ratio (PIR), educational level, BMI, smoking status, sedentary status, diabetes, and hypertension. Age was recorded as a continuous variable in years. Race had five categories: Mexican American, other Hispanic, White, Black, and other races. PIR was determined by the family income to poverty ratio (INDFMPIR), with < 2 indicating low income, > 3 indicating high income, and intermediate indicating normal income. Education was categorized as less than high school, high school graduate or equivalent, and college or higher education. BMI was measured continuously. Smoking status was dichotomized using serum cotinine concentrations, with > 14 ng/mL indicating a long smoking history [17]. 

Sedentary status was categorized by sitting time. Hypertension diagnosis followed the 2017 American Heart Association recommendations, considering antihypertensive medication use and questionnaire responses. Diabetes diagnosis adhered to American Diabetes Association criteria: glycated hemoglobin > 5.7% or 6.5%, fasting blood glucose > 100/126 mg/dL, physician-diagnosed diabetes, or impaired glucose tolerance from the questionnaire.

### WQS regression and RCS analysis

WQS regression is a statistical model for the multiple regression of high-dimensional data sets that is commonly used for environmental exposures [18]. We utilized the gWQS package to assess the overall impact of blood cells on NAFLD and performed logistic regression to analyze the results. Additionally, RCS analysis was conducted to explore linear correlations between blood cell components and NAFLD [19]. Most regression models assume a linear relationship between independent and dependent variables. The RCS, an extension of the regression spline, maintains linearity in two intervals at each end of the independent variable data range. For assessing the correlation in factors influencing NAFLD onset, RCS analysis was conducted using functions from the RMS package.

### MR analysis

Summary statistics were retrieved and obtained from OpenGWAS (https://gwas.mrcieu.ac.uk/), comprising blood cells, circulating cytokines, and growth factors associated with NAFLD GWAS summary data [20–22]. GWAS data comprised routine blood indices from 173,480 individuals of European descent in the UK Biobank and the INTERVAL study conducted by Astle et al. The analysis encompassed 36 indicators associated with red blood cells, white blood cells, platelets, and other related factors. Adjusted for age, sex, and BMI, GWAS data for 41 peripheral circulating cytokines and growth factors were sourced from the Ahola-Olli et al. study, involving 8293 Finnish individuals in the Cardiovascular Risk Study of Young Finns and FINRISK studies (FINRISK 2002 and FINRISK 1997) [21]. GWAS data for NAFLD were acquired from the FinnGen Biobank, which comprises 218,792 samples of European ancestry [20]. All participants provided informed consent in all the corresponding original studies. All data used in this work are publicly available from studies with relevant participant consent and ethical approval. Ethical approval from an institutional review board was not necessary for the present study as only publicly available summary level data was used. Instrumental variables (IVs) were selected based on criteria (*p* < 5e-08, r² < 0.001, and kb distance > 10,000). Single nucleotide polymorphisms (SNPs) with *p* > 5e-08 in NAFLD outcome variables were excluded post data integration. The genetic variant must be strongly associated with the exposure of interest. This means the variant should significantly influence the levels or presence of the risk factor being studied. If the genetic variant does not have a strong association with the exposure, it cannot be a reliable instrument.The genetic variant must be independent of confounders. This assumption requires that the genetic variant is not associated with any confounding factors that could influence both the exposure and the outcome. This is often referred to as the assumption of no pleiotropy, meaning the genetic variant affects the outcome only through its effect on the exposure, not through other pathways.The genetic variant affects the outcome only through the exposure. This means there should be no direct pathway from the genetic variant to the outcome that bypasses the exposure. Any other pathway (except through the exposure) by which the genetic variant could affect the outcome would violate this assumption and potentially lead to biased results.The TwoSampleMR R package (version 0.5.6) in R (version 4.3.1) was used to conduct all MR analyses.TwoSampleMR package facilitated expose-mediator and expose-outcome two-sample analyses [23]. Multivariate Mendelian analysis assessed outcomes, including positive exposure and mediator variables. Mediating effects were analyzed using these results. Weak instrumental variables (IVs) were identified (F-statistic < 10) and excluded from subsequent analysis. Heterogeneity and sensitivity analyses used the TwoSampleMR package. If inverse-variance weighted (IVW) estimates were significant (*p* < 0.05) without pleiotropy evidence, they were considered causal. Multivariate Mendelian randomization (MVMR), an expansion of MR, determined the effects of multiple exposures on NAFLD using correlated genetic variants. Direct effects of blood cell indicators, circulating cytokines, and growth factors on NAFLD were obtained. The blood cell indicators → circulating cytokines and growth factors → NAFLD pathway mediating effect was determined. The effect of blood cells on circulating cytokines and growth factors was analyzed using the equation:

$${\beta _{\text{M}}}\,{\text{ = }}{\beta _{\text{A}}}{\text{x}}{\beta _{\text{B}}}$$

$${\text{S}}{{\text{E}}_{\text{M}}}\, = \,\sqrt {{{{\text{(}}{\beta _{\text{A}}}{\text{x}}S{E_{\text{B}}}{\text{)}}}^2} + {{{\text{(}}{\beta _{\text{B}}}{\text{x}}S{E_{\text{A}}}{\text{)}}}^2} + S{E_{\text{A}}}^2{\text{x}}S{E_{\text{B}}}^2} $$

where β_M_ is the effect value of the mediating effect, β_A_ is the MR effect value of blood cell indicators on circulating cytokines and growth factors, β_B_ is the direct effect value of circulating cytokines and growth factors on NAFLD (obtained by multivariate MR), SE_M_ is the standard error of the mediating effect, SE_A_ is the standard error of the MR analysis of blood cell indicators on circulating cytokines and growth factors, and SE_B_ is the standard error of MR analysis of circulating cytokines and growth factors on NAFLD.

### Statistical analysis

R software (version 4.3.1) was employed for all data computations and statistical analyses. Continuous variables are expressed as medians (interquartile ranges) and were tested using the Wilcoxon rank-sum test. Categorical variables, presented as frequencies, underwent analysis using the chi-square test. Weight calculation utilized WTINT2YR, and the survey package was employed for weighted logistic and linear regressions. Significance for *p* was assumed if not otherwise specified.

## Results

### Baseline characteristics of study participants

We utilized NHANES 2017–2018 data, including 4,198 participants meeting inclusion criteria, grouped by NAFLD presence or absence. Table 1 summarized clinical characteristics, revealing significant differences in sedentary behavior, BMI, race, sex, age, diabetes mellitus, hypertension, and 14 blood cell variables (*p* < 0.05), including LBXWBCSI, LBXHGB, LBXPLTSI, LBXEOPCT, LBDLYMNO, LBDMONO, LBDNENO, LBDEONO, LBXRBCSI, LBXHCT, LBXRDW, LBXMCVSI, LBXMCHSI, and LBDBANO.

**Table 1:** Baseline comparisons of study participants

Characteristic	Overall, *N* = 4198 ^1^	normal, *N* = 2353 ^1^	NAFLD, *N* = 1845 ^1^	*p* ^2^
LBXWBCSI	7.00 (5.80, 8.50)	6.70 (5.50, 8.10)	7.50 (6.30, 9.00)	**< 0.001**
LBXLYPCT	31 (25, 36)	31 (25, 37)	30 (25, 35)	0.062
LBXMOPCT	7.90 (6.70, 9.30)	7.90 (6.70, 9.30)	8.00 (6.70, 9.30)	0.8
LBXNEPCT	58 (52, 64)	57 (51, 64)	58 (53, 64)	0.12
LBXEOPCT	2.30 (1.50, 3.40)	2.20 (1.40, 3.30)	2.30 (1.60, 3.52)	**0.010**
LBXBAPCT	0.70 (0.60, 1.00)	0.70 (0.50, 1.00)	0.80 (0.60, 1.00)	0.13
LBDLYMNO	2.10 (1.70, 2.60)	2.10 (1.60, 2.50)	2.20 (1.80, 2.70)	**< 0.001**
LBDMONO	0.60 (0.50, 0.70)	0.50 (0.40, 0.60)	0.60 (0.50, 0.70)	**< 0.001**
LBDNENO	4.00 (3.10, 5.20)	3.80 (2.90, 4.90)	4.30 (3.40, 5.50)	**< 0.001**
LBDEONO	0.20 (0.10, 0.20)	0.10 (0.10, 0.20)	0.20 (0.10, 0.30)	**< 0.001**
LBDBANO				**< 0.001**
0	1,990 (47%)	1,224 (53%)	766 (40%)	
0.1	2,174 (52%)	1,112 (46%)	1,062 (60%)	
0.2	33 (0.7%)	17 (0.7%)	16 (0.7%)	
0.3	1 (< 0.1%)	0 (0%)	1 (< 0.1%)	
LBXRBCSI	4.76 (4.45, 5.08)	4.67 (4.37, 5.01)	4.86 (4.58, 5.17)	**< 0.001**
LBXHGB	14.30 (13.30, 15.20)	14.00 (13.20, 15.00)	14.50 (13.60, 15.40)	**< 0.001**
LBXHCT	42.3 (39.7, 44.8)	41.8 (39.3, 44.2)	43.0 (40.3, 45.6)	**< 0.001**
LBXMCVSI	89.1 (85.9, 92.1)	89.6 (86.5, 92.5)	88.5 (85.2, 91.3)	**< 0.001**
LBXMCHSI	30.10 (28.90, 31.20)	30.30 (29.10, 31.30)	29.80 (28.70, 31.00)	**0.001**
LBXMC	33.70 (33.20, 34.20)	33.70 (33.10, 34.20)	33.70 (33.20, 34.20)	0.13
LBXRDW	13.40 (13.00, 14.00)	13.30 (12.90, 13.90)	13.50 (13.06, 14.10)	**0.002**
LBXPLTSI	238 (204, 281)	235 (201, 277)	243 (208, 284)	**0.003**
LBXMPSI	8.20 (7.60, 8.80)	8.20 (7.60, 8.80)	8.20 (7.60, 8.80)	> 0.9
**Hypertension**	1,569 (31%)	696 (22%)	873 (44%)	**< 0.001**
**Sedentary**				**0.015**
< 3 h	1,341 (27%)	791 (29%)	550 (24%)	
3–6 h	1,533 (37%)	850 (37%)	683 (38%)	
> 6 h	1,324 (35%)	712 (34%)	612 (38%)	
**Smoker**	934 (22%)	567 (23%)	367 (20%)	0.089
**BMI**	29 (25, 34)	26 (23, 30)	33 (29, 37)	**< 0.001**
**RIP**				> 0.9
low	1,661 (28%)	921 (28%)	740 (28%)	
normal	1,123 (24%)	632 (24%)	491 (24%)	
high	1,414 (48%)	800 (48%)	614 (48%)	
**Education**				0.11
Below high school	347 (3.5%)	168 (3.1%)	179 (4.1%)	
High school	1,434 (34%)	793 (32%)	641 (36%)	
Above high school	2,417 (63%)	1,392 (65%)	1,025 (60%)	
**Race**				**< 0.001**
Non-Hispanic White	1,441 (63%)	788 (63%)	653 (63%)	
Non-Hispanic Black	942 (11%)	615 (13%)	327 (8.4%)	
Mexican American	583 (8.9%)	238 (6.5%)	345 (12%)	
Other Hispanic	403 (7.0%)	227 (7.5%)	176 (6.2%)	
Other Race	829 (10%)	485 (11%)	344 (10%)	
**Sex**				**< 0.001**
Male	2,039 (48%)	1,031 (43%)	1,008 (55%)	
Female	2,159 (52%)	1,322 (57%)	837 (45%)	
**Age**	48.0 (33.0, 61.0)	44.0 (31.0, 59.0)	52.0 (38.0, 63.0)	**< 0.001**
**Diabetes**				**< 0.001**
normal	1,883 (51%)	1,323 (62%)	560 (34%)	
prediabetes	1,495 (36%)	761 (31%)	734 (42%)	
diabetes	820 (13%)	269 (6.3%)	551 (23%)	

^1^Median (IQR); n (unweighted) (%)

^2^Wilcoxon rank-sum test for complex survey samples; chi-squared test with Rao & Scott’s second-order correction

LBXWBCSI: White blood cell count (1000 cells/uL); LBXLYPCT: Lymphocyte percent (%); LBXMOPCT: Monocyte percent (%); LBXNEPCT: Segmented neutrophils percent (%); LBXEOPCT: Eosinophils percent (%); LBXBAPCT: Basophils percent (%); LBDLYMNO; Lymphocyte number (1000 cells/uL); LBDMONO; Monocyte number (1000 cells/uL); LBDNENO; Segmented neutrophils num (1000 cell/uL); LBDEONO; Eosinophils number (1000 cells/uL); LBDBANO; Basophils number (1000 cells/uL); LBXRBCSI; Red blood cell count (million cells/uL); LBXHGB; Hemoglobin (g/dL); LBXHCT; Hematocrit (%); LBXMCVSI; Mean cell volume (fL); LBXMCHSI; Mean cell hemoglobin (pg); LBXMC; MCHC (g/dL); LBXRDW; Red cell distribution width (%); LBXPLTSI; Platelet count SI (1000 cells/uL); LBXMPSI; Mean platelet volume (fL)

### Association between blood cells and NAFLD

To depict the association between the 14 significant blood cell parameters (LBXWBCSI, LBXRBCSI, LBXHGB, LBXPLTSI, LBXEOPCT, LBDMONO, LBDLYMNO, LBDEONO, LBDBANO, LBDNENO, LBXHCT, LBXMCVSI, LBXMCHSI, and LBXRDW) and NAFLD, two multivariate logistic regression models were constructed. Model 1 adjusted for significant baseline NAFLD comparison covariates (hypertension, sedentary status, BMI, race, sex, age, and diabetes). Model 2 included all Model 1 covariates and additional ones (smoking status, RIP, and educational level). Results in Table 2 demonstrated that 10 indicators (LBXWBCSI, LBXHGB, LBXPLTSI, LBDLYMNO, LBDMONO, LBDNENO, LBXRBCSI, LBXHCT, LBXRDW, and LBDBANO) are all independent influencers for NAFLD. Except LBXRDW exhibited a negative correlation with NAFLD (Model 1: OR = 0.466, 95% CI: 0.239–0.900, *p* = 0.024; Model 2: OR = 0.452, 95% CI: 0.231–0.878, *p* = 0.020), while others showed independent risk factors.

**Table 2:** Multivariate logistic regression of blood cells on NAFLD (all participants)

		Model 1		Model 2	
	Total	OR[95%CI]	*p*	**OR[95%CI]**	*p*
LBXWBCSI	4,198	1.545[1.258, 1.900]	**< 0.001**	1.574[1.275, 1.945]	**< 0.001**
LBXEOPCT	4,198	1.014[0.910, 1.131]	0.798	1.016[0.911, 1.133]	0.774
LBDLYMNO	4,198	2.046[1.627, 2.579]	**< 0.001**	2.080[1.650, 2.630]	**< 0.001**
LBDMONO	4,198	1.788[1.166, 2.753]	**0.008**	1.801[1.171, 2.784]	**0.008**
LBDNENO	4,198	1.187[1.001, 1.409]	**0.049**	1.192[1.001, 1.419]	**0.048**
LBDEONO	4,198	1.296[0.856, 1.963]	0.221	1.306[0.861, 1.981]	0.209
LBXRBCSI	4,198	5.259[2.725, 10.187]	**< 0.001**	5.377[2.781, 10.431]	**< 0.001**
LBXHGB	4,198	4.029[2.275, 7.183]	**< 0.001**	4.110[2.312, 7.360]	**< 0.001**
LBXHCT	4,198	3.274[1.806, 5.965]	**< 0.001**	3.324[1.827, 6.076]	**< 0.001**
LBXMCVSI	4,198	0.509[0.245, 1.053]	0.069	0.491[0.234, 1.024]	0.059
LBXMCHSI	4,198	0.993[0.536, 1.843]	0.982	0.978[0.524, 1.827]	0.944
LBXRDW	4,198	0.466[0.239, 0.900]	**0.024**	0.452[0.231, 0.878]	**0.020**
LBXPLTSI	4,198	1.237[1.012, 1.513]	**0.039**	1.231[1.007, 1.508]	**0.044**
LBDBANO	4,198	5.694[2.056, 15.790]	**0.001**	5.739[2.053, 16.065]	**0.001**

LBXWBCSI: White blood cell count (1000 cells/uL); LBXLYPCT: Lymphocyte percent (%); LBXMOPCT: Monocyte percent (%); LBXNEPCT: Segmented neutrophils percent (%); LBXEOPCT: Eosinophils percent (%); LBXBAPCT: Basophils percent (%); LBDLYMNO; Lymphocyte number (1000 cells/uL); LBDMONO; Monocyte number (1000 cells/uL); LBDNENO; Segmented neutrophils num (1000 cell/uL); LBDEONO; Eosinophils number (1000 cells/uL); LBDBANO; Basophils number (1000 cells/uL); LBXRBCSI; Red blood cell count (million cells/uL); LBXHGB; Hemoglobin (g/dL); LBXHCT; Hematocrit (%); LBXMCVSI; Mean cell volume (fL); LBXMCHSI; Mean cell hemoglobin (pg); LBXMC; MCHC (g/dL); LBXRDW; Red cell distribution width (%); LBXPLTSI; Platelet count SI (1000 cells/uL); LBXMPSI; Mean platelet volume (fL)

### WQS analysis and RCS analysis of blood cells for NAFLD

**Fig. 2:**
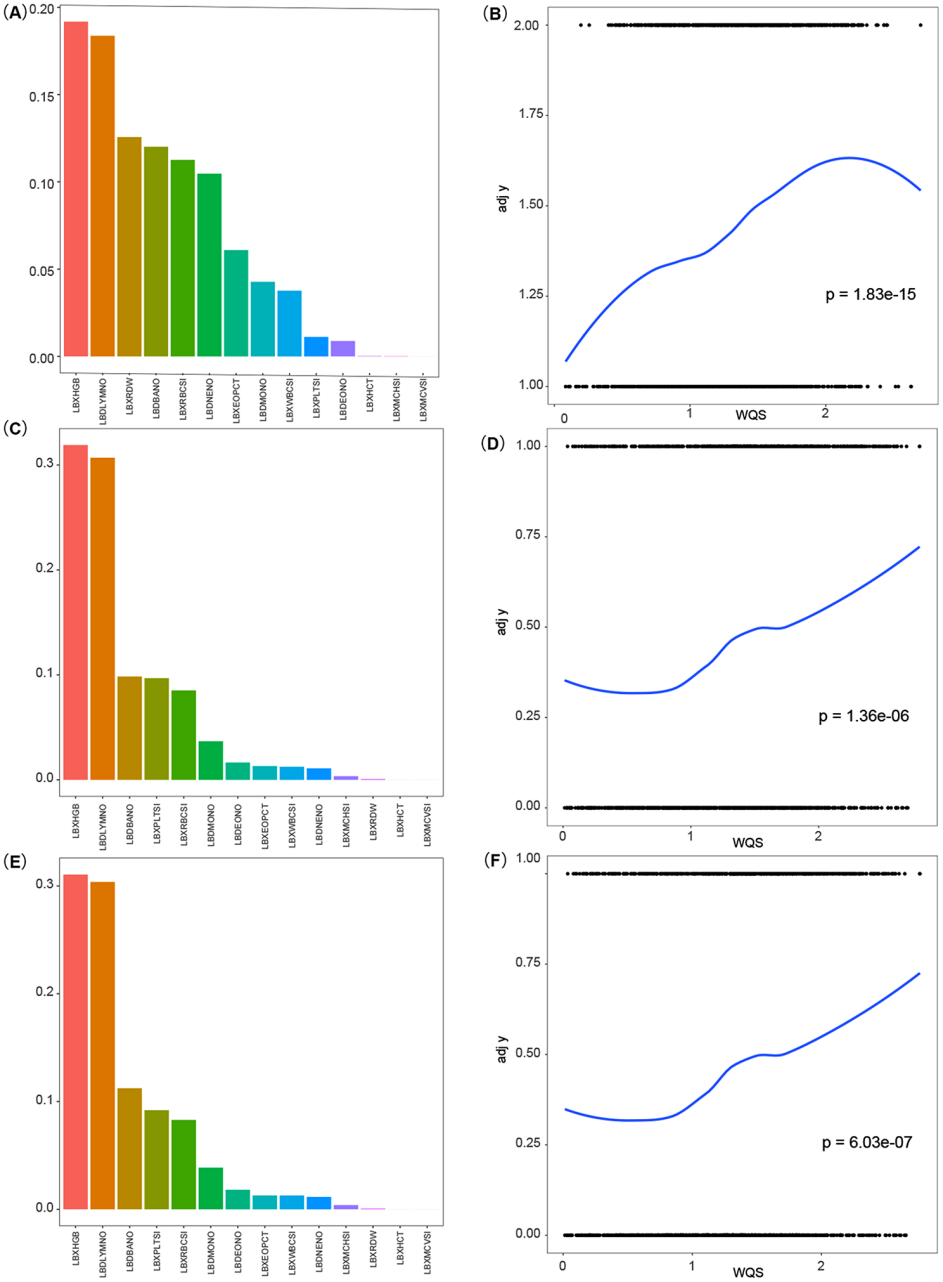
WQS analysis of blood cells for nonalcoholic fatty liver disease. A: Unadjusted impact of blood cells on NAFLD; B: Unadjusted association between blood cell WQS index and NAFLD; C: Partially corrected impact of blood cells on NAFLD; D: Partially corrected association between blood cell WQS index and NAFLD; E: Fully corrected impact of blood cells on NAFLD; F: Fully corrected association between blood cell WQS index and NAFLD. (WQS: Weighted Quantile Sum)

**Fig. 3:**
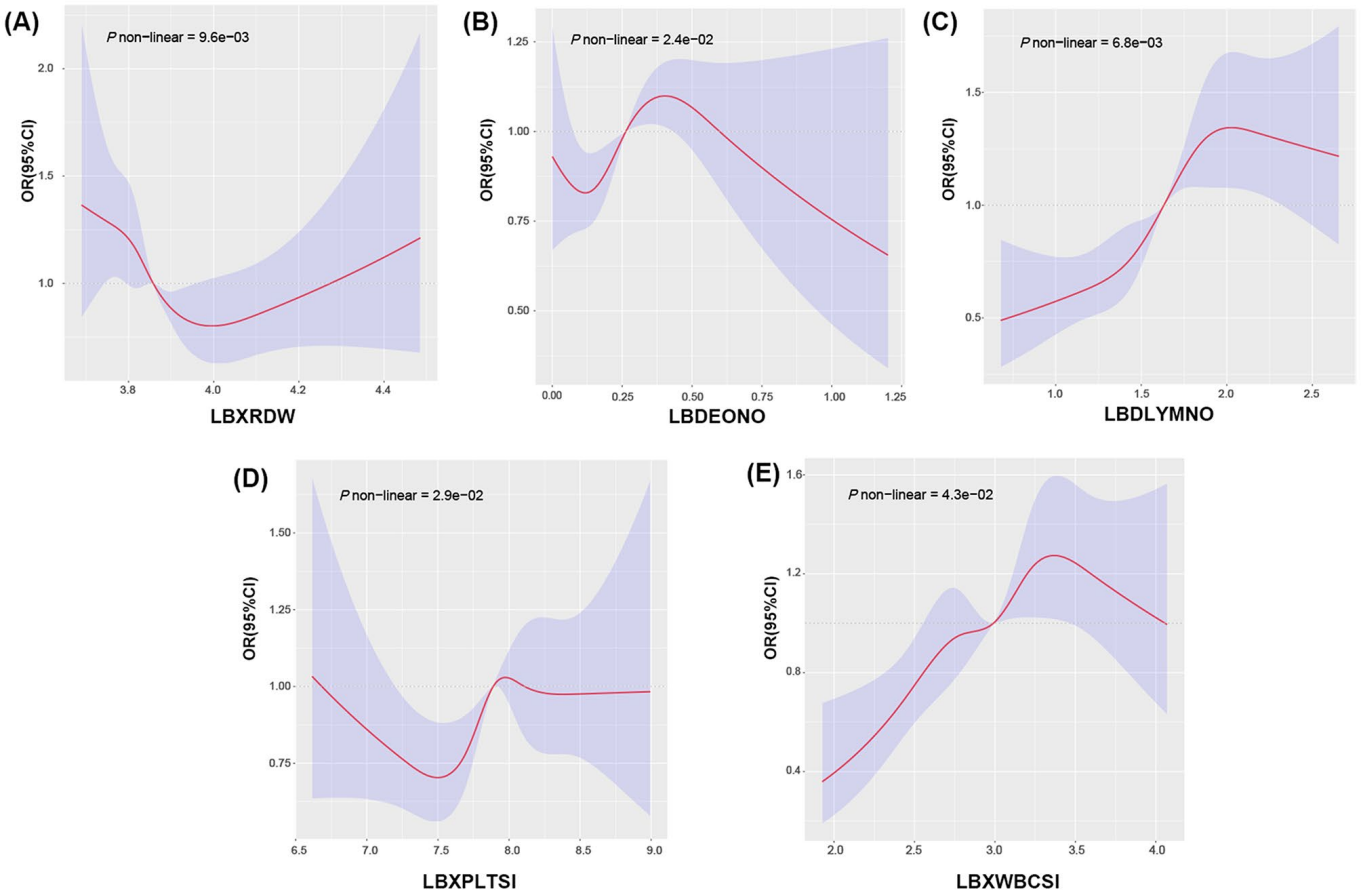
Restricted cubic spline analysis between blood cell and NAFLD. A: Restricted cubic spline analysis of LBXRDW and NAFLD; B: Restricted cubic spline analysis of LBDEONO and NAFLD; C: Restricted cubic spline analysis of LBDLYMNO and NAFLD; D: Restricted cubic spline analysis of LBXPLTSI and NAFLD; E: Restricted cubic spline analysis of LBXWBCSI and NAFLD; (LBXWBCSI: White blood cell count, LBDEONO:Eosinophils number, LBDLYMNO: Lymphocyte number, LBXRDW: Red cell distribution width, LBXPLTSI: Platelet count SI)

We conducted a WQS analysis to validate the relationship and contribution of the 14 significant blood cell parameters (shown in Table 1) to NAFLD. Results without adjustment for WQS showed a positive association (*p* = 1.83e-15) between total blood cell indices and NAFLD (Fig. 2B). After adjusting for significant baseline covariates (hypertension, sedentary behavior, BMI, race, sex, age, and diabetes) and all covariates, a positive association remained in two of the above models (Fig. 2D, F; *p* = 1.36e-06, *p* = 6.03e-07). LBXHGB and LBDLYMNO consistently contributed the most. Nonlinear relationships were assessed using RCS for the 14 parameters, except LBDBANO, which is categorical. Significant nonlinear relationships were observed for LBXRDW, LBDEONO, LBDLYMNO, LBXPLTSI, and LBXWBCSI (Fig. 3).

### MR analysis

**Fig. 4:**
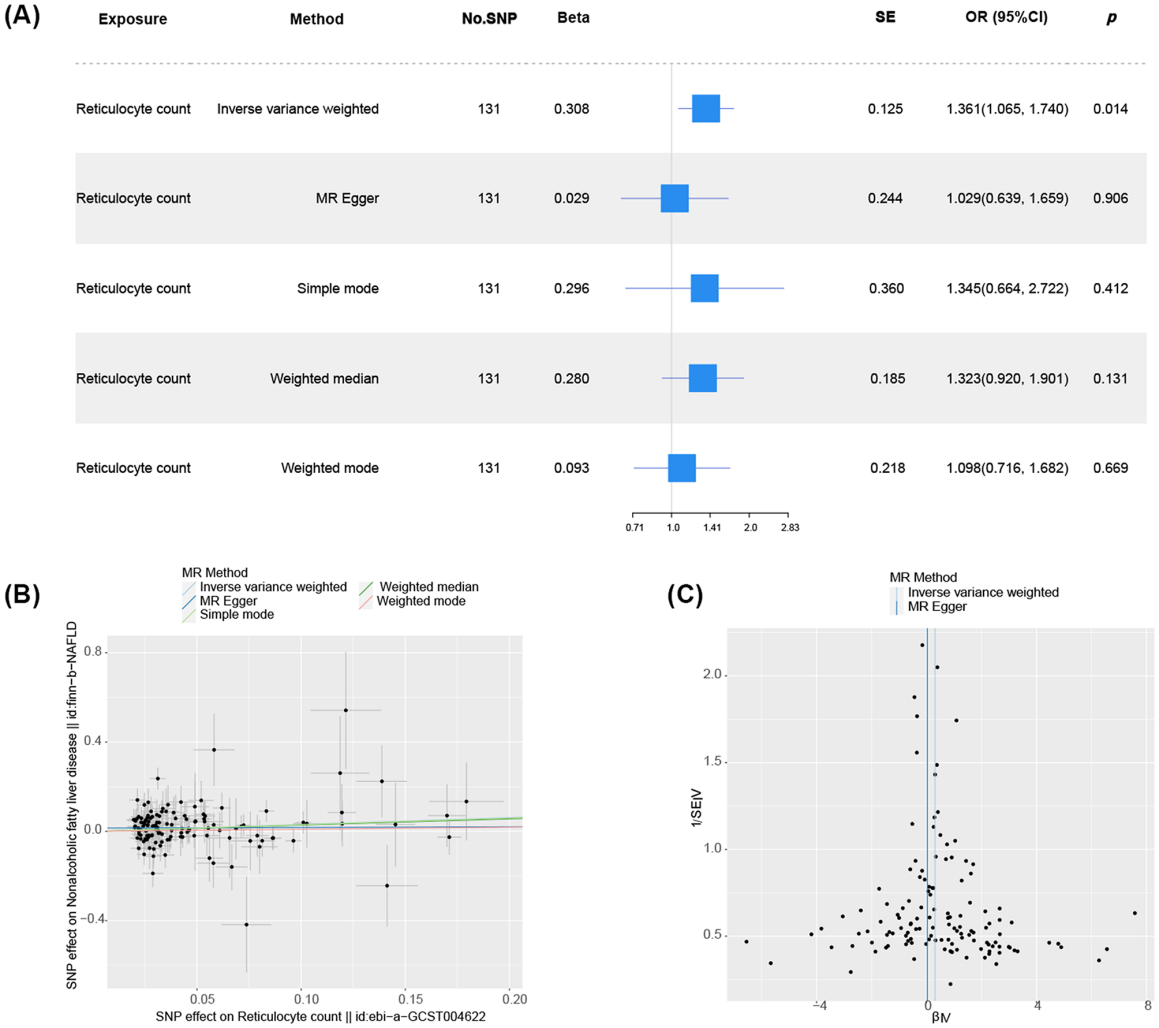
MR result of Reticulocyte count on NAFLD. A: Causal Forest Plot of Reticulocyte count on NAFLD; B: Causal Scatter Plot of Reticulocyte count on NAFLD; C: Heterogeneity Analysis Funnel Plot of Reticulocyte count on NAFLD; IVW: Inverse-variance weighted

To explore the causal link between blood cells (Detailed information on IVs shown in Table S4) and NAFLD, we conducted an MR analysis using blood cell-related indicators as exposure, NAFLD as the outcome, and circulating cytokines and growth factors as mediators. Two-sample MR with SNPs as IVs, utilizing IVW methods, revealed a significant positive causal relationship between reticulocyte count and NAFLD (IVW, OR = 1.361, 95% CI: 1.065–1.740, *p* = 0.014)(complete results in Table S1). The MR–Egger test, weighted median, simple mode, and weighted mode tests also showed directional coherence (Fig. 4A). Heterogeneity assessment and funnel plot analysis suggested no observed heterogeneity (*p* of Q-value = 0.125) and correct IV selection (Fig. 4C). Egger’s method indicated no pleiotropy (Table 3). Steiger directionality test and leave-one-out test supported no reverse causality and stability of IVW results (Table S2). Univariable Mendelian randomization and MVMR revealed that circulating cytokine and growth factors were not significant mediator (complete results in Table S3).

**Table 3:** Heterogeneity and Pleiotropy test between Reticulocyte count and NAFLD

	Exposure	Outcome	Method	Q	Q_df	Q_p
**Heterogeneity test**	Reticulocyte count	NAFLD	MR Egger	146.714	129	0.136
	Reticulocyte count	NAFLD	IVW	148.750	130	0.125
	**Exposure**	**Outcome**	**Egger_intercept**		**SE**	***p***
**Pleiotropy test**	Reticulocyte count	NAFLD	0.0146		0.011	0.183

## Discussion

In this study, we investigated the correlation between various blood cell indicators and NAFLD, combining a 2017–2018 NHANES cross-sectional study with a two-sample MR analysis. Ours is the first study to explore this correlation by integrating NHANES data and extensive genetic analysis. Ultimately, we established a connection between blood cell indicators and an increased risk of NAFLD.

The inflammatory response is associated with NAFLD based on the hypothesis of multiple parallel hits that connects intestinal and adipocyte cells [24]. Elevated white blood cell counts and hemoglobin levels are independently correlated with NAFLD, which might reflect subclinical, low-grade systemic inflammation [25]. Platelets act as mediators of thrombosis and play a crucial role in NAFLD progression as they promote a pro-thrombotic and pro-inflammatory environment [26]. Increased binding of leukocytes and platelets, through potentially mechanisms such as neutrophil extracellular traps, may exacerbate inflammation and contribute to the development and progression of NAFLD [27]. Blood cell indicators could be promising biomarkers for NAFLD monitoring in the clinic. However, the overall connection between blood cell parameters, the significance of each indicator, and whether a causal relationship exists with NAFLD remain unclear.

In our observational study, WQS analyses of 14 variables showed a significant positive correlation with NAFLD incidence. Hemoglobin and lymphocyte counts carried most of the weights, indicating their significant influence. Lymphocytes are a very important immune-related cell class, and the finding of a non-linear relationship highlights the significance and complexity of lymphocytes in NAFLD, which may be closely related to the different subtypes of lymphocytes and their secreted factors [28]. Specifically, interleukin-6 possesses both inflammation-promoting and inflammation-suppressing attributes [29], while an explicit mechanism requires further study. Given its significance, the lymphocyte count may be an appropriate indicator in blood routine examination to monitor the course and progression of NAFLD. Observational studies may be biased by unmeasured or uncontrolled factors that affect the accuracy of results and indicate reverse causation, so it is important to explore the relationship between blood cell indicators and NAFLD risk from a genetic perspective.

In the MR analysis, no causal link was found between routine blood cell indicators and NAFLD. However, a surprising positive association was observed with reticulocyte count. To establish a robust correlation, we selected IVs strongly linked to hematological markers from the GWAS summary that meet a genome-wide significance threshold (*p* < 5 e - 08) after linkage disequilibrium was removed, ensuring more reliable and independent outcomes [30]. Through pleiotropy and heterogeneity examinations, IVs were found to directly impact the onset of NAFLD through reticulocytes. However, the study did not establish a mechanism for how reticulocytes could cause NAFLD or liver-related metabolic disorders. Circulating cytokines and growth factors were not found to be mediators, suggesting unknown reticulocyte mediators in NAFLD pathophysiology.Therefore, further investigation is needed to understand these mechanisms.

The main advantage of this study is the use of the NHANES database and MR analysis to explore the association between blood cells parameters and NAFLD risk. This combination of methods improves the reliability of the findings. Our MR analyses, which included extensive data, provided sufficient statistical power to estimate the correlation between blood cell indicators and NAFLD. However, a few limitations of our study should be acknowledged.Information on NAFLD was obtained from questionnaire and CAP. Although CAP is a useful non-invasive tool to assess NAFLD, it still has limitations compared to biopsy, especially in clarifying the severity of NAFLD, but is more applicable in studies with large-scale populations. Due to limitations in study sample size, variable details and data access, lipid-lowering medications and alcohol consumption were excluded as covariates, making it impossible to adequately assess their impact in this study, and limiting the exclusion of certain alcohol-related diseases, such as alcohol-induced chronic pancreatitis, alcoholic fatty liver. Although diabetes was covariate adjusted in our study sample, the strong association between diabetes mellitus and NAFLD cannot be disregarded. So in future studies we will include larger samples for a more comprehensive study. NHANES data included individuals with missing blood cell information, which may have introduced selection bias, although the consistency of the distribution of participants suggested otherwise. RCS analyses explored nonlinear associations, but limited statistical power might have masked substantial connections. Our findings were based on European and USA populations, which limited generalizability to other ethnicities, especially if the genetic variants studied are not representative of the broader population. MR studies may oversimplify relationships and not capture full causality for complex traits influenced by many genetic and environmental factors. Although we took particular care to use a variety of statistical methods, including heterogeneity analyses, Egger’s method for detecting pleiotropy, and sensitivity analyses to increase the robustness of the results, but it is difficult to thoroughly address all hypothetical challenges, and the possibility of residual confounding or pleiotropic effects may still exist.The study concluded that a positive causal connection exists between reticulocyte count and the incidence of NAFLD and only identified concurrent phenomena related to liver disease [31] and inflammatory bowel diseases [32]; however, the mechanism of the interaction has not been elucidated, and further prospective and mechanistic studies are required for validation.

## Conclusions

Our study suggests that certain hematological markers may pose independent NAFLD risk factors, although lacking a causal link with its incidence. A positive causal relationship was identified with reticulocyte count and NAFLD occurrence. These findings lay a foundation for monitoring NAFLD pathogenesis, but detailed mechanisms underlying this correlation require further investigation.

### Electronic supplementary material

Below is the link to the electronic supplementary material.

Supplementary Material 1

Supplementary Material 2

Supplementary Material 3

Supplementary Material 4

## Data Availability

The database is publicly available. All data generated or analysed during this study are included in this published article and its supplementary information files.

## Abbreviations

CI: confidence interval
GWAS: genome-wide association studies
IVW: inverse-variance weighted
LUTE: liver ultrasound transient elastography
MR: Mendelian randomization
IVs: instrumental variables
MVMR: multivariate Mendelian randomization
NAFLD: nonalcoholic fatty liver disease
MASLD: metabolic dysfunction-associated steatotic liver disease
NHANES: National Health and Nutrition Examination Survey
OR: odds ratio
RCS: restricted cubic spline
WQS: weighted quantile sum
