# Supplementary material for: Blood cell parameters and risk of nonalcoholic fatty liver disease: a comprehensive Mendelian randomization study
Source: BMC Med Genomics. 2024 Apr 23;17:102. doi: 10.1186/s12920-024-01879-7 (PMC11040836; doi:10.1186/s12920-024-01879-7)
Supplement: Supplementary file 1 — Supplementary Material 1 [file 12920_2024_1879_MOESM1_ESM.doc]

**Table S1**

| **MR blood cell indicators -NAFLD causal effect estimation** | | | | | | | | |
| --- | --- | --- | --- | --- | --- | --- | --- | --- |
| **id.exposure** | **id.outcome** | **Exposure** | **Outcome** | **Method** | **No.SNP** | **Beta** | **ES** | **p** |
| ebi-a-GCST004599 | finn-b-NAFLD | Mean platelet volume | Nonalcoholic fatty liver disease | Inverse variance weighted | 179 | 0.002 | 0.086 | 0.981 |
| ebi-a-GCST004600 | finn-b-NAFLD | Eosinophil percentage of white cells | Nonalcoholic fatty liver disease | Inverse variance weighted | 125 | -0.148 | 0.132 | 0.264 |
| ebi-a-GCST004601 | finn-b-NAFLD | Red blood cell count | Nonalcoholic fatty liver disease | Inverse variance weighted | 145 | 0.032 | 0.114 | 0.777 |
| ebi-a-GCST004602 | finn-b-NAFLD | Mean corpuscular volume | Nonalcoholic fatty liver disease | Inverse variance weighted | 172 | 0.012 | 0.088 | 0.893 |
| ebi-a-GCST004603 | finn-b-NAFLD | Platelet count | Nonalcoholic fatty liver disease | Inverse variance weighted | 169 | 0.096 | 0.111 | 0.388 |
| ebi-a-GCST004604 | finn-b-NAFLD | Hematocrit | Nonalcoholic fatty liver disease | Inverse variance weighted | 91 | 0.247 | 0.173 | 0.154 |
| ebi-a-GCST004605 | finn-b-NAFLD | Mean corpuscular hemoglobin concentration | Nonalcoholic fatty liver disease | Inverse variance weighted | 65 | 0.133 | 0.174 | 0.442 |
| ebi-a-GCST004606 | finn-b-NAFLD | Eosinophil counts | Nonalcoholic fatty liver disease | Inverse variance weighted | 142 | -0.090 | 0.122 | 0.463 |
| ebi-a-GCST004607 | finn-b-NAFLD | Plateletcrit | Nonalcoholic fatty liver disease | Inverse variance weighted | 182 | 0.054 | 0.104 | 0.603 |
| ebi-a-GCST004608 | finn-b-NAFLD | Granulocyte percentage of myeloid white cells | Nonalcoholic fatty liver disease | Inverse variance weighted | 129 | 0.182 | 0.126 | 0.148 |
| ebi-a-GCST004609 | finn-b-NAFLD | Monocyte percentage of white cells | Nonalcoholic fatty liver disease | Inverse variance weighted | 151 | -0.147 | 0.100 | 0.145 |
| ebi-a-GCST004610 | finn-b-NAFLD | White blood cell count | Nonalcoholic fatty liver disease | Inverse variance weighted | 130 | 0.122 | 0.141 | 0.384 |
| ebi-a-GCST004611 | finn-b-NAFLD | High light scatter reticulocyte count | Nonalcoholic fatty liver disease | Inverse variance weighted | 146 | 0.181 | 0.120 | 0.130 |
| ebi-a-GCST004612 | finn-b-NAFLD | High light scatter reticulocyte percentage of red cells | Nonalcoholic fatty liver disease | Inverse variance weighted | 153 | 0.120 | 0.118 | 0.310 |
| ebi-a-GCST004613 | finn-b-NAFLD | Sum neutrophil eosinophil counts | Nonalcoholic fatty liver disease | Inverse variance weighted | 113 | 0.150 | 0.166 | 0.367 |
| ebi-a-GCST004614 | finn-b-NAFLD | Granulocyte count | Nonalcoholic fatty liver disease | Inverse variance weighted | 118 | 0.144 | 0.159 | 0.366 |
| ebi-a-GCST004615 | finn-b-NAFLD | Hemoglobin concentration | Nonalcoholic fatty liver disease | Inverse variance weighted | 97 | 0.216 | 0.167 | 0.197 |
| ebi-a-GCST004616 | finn-b-NAFLD | Platelet distribution width | Nonalcoholic fatty liver disease | Inverse variance weighted | 63 | 0.298 | 0.176 | 0.090 |
| ebi-a-GCST004617 | finn-b-NAFLD | Eosinophil percentage of granulocytes | Nonalcoholic fatty liver disease | Inverse variance weighted | 127 | -0.033 | 0.134 | 0.804 |
| ebi-a-GCST004618 | finn-b-NAFLD | White blood cell count (basophil) | Nonalcoholic fatty liver disease | Inverse variance weighted | 61 | -0.184 | 0.241 | 0.446 |
| ebi-a-GCST004619 | finn-b-NAFLD | Reticulocyte fraction of red cells | Nonalcoholic fatty liver disease | Inverse variance weighted | 139 | 0.186 | 0.127 | 0.143 |
| ebi-a-GCST004620 | finn-b-NAFLD | Sum basophil neutrophil counts | Nonalcoholic fatty liver disease | Inverse variance weighted | 109 | 0.207 | 0.164 | 0.208 |
| ebi-a-GCST004621 | finn-b-NAFLD | Sum basophil neutrophil counts | Nonalcoholic fatty liver disease | Inverse variance weighted | 124 | -0.190 | 0.120 | 0.114 |
| **ebi-a-GCST004622** | **finn-b-NAFLD** | **Reticulocyte count** | **Nonalcoholic fatty liver disease** | **Inverse variance weighted** | **131** | **0.308** | **0.125** | **0.014** |
| ebi-a-GCST004623 | finn-b-NAFLD | Neutrophil percentage of granulocytes | Nonalcoholic fatty liver disease | Inverse variance weighted | 122 | -0.056 | 0.138 | 0.682 |
| ebi-a-GCST004624 | finn-b-NAFLD | Sum eosinophil basophil counts | Nonalcoholic fatty liver disease | Inverse variance weighted | 143 | 0.062 | 0.143 | 0.664 |
| ebi-a-GCST004625 | finn-b-NAFLD | Monocyte count | Nonalcoholic fatty liver disease | Inverse variance weighted | 141 | -0.153 | 0.110 | 0.164 |
| ebi-a-GCST004626 | finn-b-NAFLD | Myeloid white cell count | Nonalcoholic fatty liver disease | Inverse variance weighted | 116 | 0.161 | 0.141 | 0.252 |
| ebi-a-GCST004627 | finn-b-NAFLD | Lymphocyte counts | Nonalcoholic fatty liver disease | Inverse variance weighted | 126 | 0.088 | 0.138 | 0.525 |
| ebi-a-GCST004628 | finn-b-NAFLD | Immature fraction of reticulocytes | Nonalcoholic fatty liver disease | Inverse variance weighted | 107 | 0.113 | 0.128 | 0.374 |
| ebi-a-GCST004629 | finn-b-NAFLD | Neutrophil count | Nonalcoholic fatty liver disease | Inverse variance weighted | 107 | 0.233 | 0.161 | 0.148 |
| ebi-a-GCST004630 | finn-b-NAFLD | Mean corpuscular hemoglobin | Nonalcoholic fatty liver disease | Inverse variance weighted | 175 | 0.054 | 0.087 | 0.536 |
| ebi-a-GCST004631 | finn-b-NAFLD | Basophil percentage of white cells | Nonalcoholic fatty liver disease | Inverse variance weighted | 49 | 0.066 | 0.238 | 0.782 |
| ebi-a-GCST004632 | finn-b-NAFLD | Lymphocyte percentage of white cells | Nonalcoholic fatty liver disease | Inverse variance weighted | 105 | 0.025 | 0.173 | 0.887 |
| ebi-a-GCST004633 | finn-b-NAFLD | Neutrophil percentage of white cells | Nonalcoholic fatty liver disease | Inverse variance weighted | 106 | -0.053 | 0.195 | 0.784 |
| ebi-a-GCST004634 | finn-b-NAFLD | Basophil percentage of granulocytes | Nonalcoholic fatty liver disease | Inverse variance weighted | 46 | -0.066 | 0.225 | 0.769 |
| **Steiger test** | | | | | | | | |
| **id.exposure** | **id.outcome** | **Exposure** | **Outcome** | **SNP_r2.**  **exposure** | **SNP_r2.**  **outcome** | **Correct_causal_direction** | **Steiger*_*p** | |
| ebi-a-GCST004622 | finn-b-NAFLD | Reticulocyte count | Nonalcoholic fatty liver disease | 0.09697297 | 0.0007578663 | TRUE | 0e+00 | |
