# Supplementary material for: Blood cell parameters and risk of nonalcoholic fatty liver disease: a comprehensive Mendelian randomization study
Source: BMC Med Genomics. 2024 Apr 23;17:102. doi: 10.1186/s12920-024-01879-7 (PMC11040836; doi:10.1186/s12920-024-01879-7)
Supplement: Supplementary file 2 — Supplementary Material 2 [file 12920_2024_1879_MOESM2_ESM.doc]

**Table S2 Leave-one-out test**

| **id.exposure** | **id.outcome** | **Exposure** | **Outcome** | **SNP** | | **Beta** | | **SE** | | **p** |
| --- | --- | --- | --- | --- | --- | --- | --- | --- | --- | --- |
| ebi-a-GCST004622 | finn-b-NAFLD | Reticulocyte count | Nonalcoholic fatty liver disease | rs644592 | 0.329 | | 0.122 | | 0.007 | |
| ebi-a-GCST004622 | finn-b-NAFLD | Reticulocyte count | Nonalcoholic fatty liver disease | rs1076872 | | 0.346 | | 0.128 | | 0.007 |
| ebi-a-GCST004622 | finn-b-NAFLD | Reticulocyte count | Nonalcoholic fatty liver disease | rs7221322 | | 0.338 | | 0.128 | | 0.008 |
| ebi-a-GCST004622 | finn-b-NAFLD | Reticulocyte count | Nonalcoholic fatty liver disease | rs1339847 | | 0.340 | | 0.130 | | 0.009 |
| ebi-a-GCST004622 | finn-b-NAFLD | Reticulocyte count | Nonalcoholic fatty liver disease | rs1036332 | | 0.325 | | 0.124 | | 0.009 |
| ebi-a-GCST004622 | finn-b-NAFLD | Reticulocyte count | Nonalcoholic fatty liver disease | rs35689703 | | 0.325 | | 0.124 | | 0.009 |
| ebi-a-GCST004622 | finn-b-NAFLD | Reticulocyte count | Nonalcoholic fatty liver disease | rs12277078 | | 0.326 | | 0.124 | | 0.009 |
| ebi-a-GCST004622 | finn-b-NAFLD | Reticulocyte count | Nonalcoholic fatty liver disease | rs4737010 | | 0.331 | | 0.127 | | 0.009 |
| ebi-a-GCST004622 | finn-b-NAFLD | Reticulocyte count | Nonalcoholic fatty liver disease | rs57467915 | | 0.325 | | 0.125 | | 0.009 |
| ebi-a-GCST004622 | finn-b-NAFLD | Reticulocyte count | Nonalcoholic fatty liver disease | rs61469632 | | 0.324 | | 0.125 | | 0.009 |
| ebi-a-GCST004622 | finn-b-NAFLD | Reticulocyte count | Nonalcoholic fatty liver disease | rs116468353 | | 0.318 | | 0.124 | | 0.010 |
| ebi-a-GCST004622 | finn-b-NAFLD | Reticulocyte count | Nonalcoholic fatty liver disease | rs74417235 | | 0.324 | | 0.127 | | 0.011 |
| ebi-a-GCST004622 | finn-b-NAFLD | Reticulocyte count | Nonalcoholic fatty liver disease | rs1505307 | | 0.318 | | 0.125 | | 0.011 |
| ebi-a-GCST004622 | finn-b-NAFLD | Reticulocyte count | Nonalcoholic fatty liver disease | rs3169166 | | 0.320 | | 0.126 | | 0.011 |
| ebi-a-GCST004622 | finn-b-NAFLD | Reticulocyte count | Nonalcoholic fatty liver disease | rs9894732 | | 0.319 | | 0.125 | | 0.011 |
| ebi-a-GCST004622 | finn-b-NAFLD | Reticulocyte count | Nonalcoholic fatty liver disease | rs7412 | | 0.318 | | 0.125 | | 0.011 |
| ebi-a-GCST004622 | finn-b-NAFLD | Reticulocyte count | Nonalcoholic fatty liver disease | rs784487 | | 0.318 | | 0.126 | | 0.011 |
| ebi-a-GCST004622 | finn-b-NAFLD | Reticulocyte count | Nonalcoholic fatty liver disease | rs35023389 | | 0.317 | | 0.125 | | 0.011 |
| ebi-a-GCST004622 | finn-b-NAFLD | Reticulocyte count | Nonalcoholic fatty liver disease | rs2968478 | | 0.318 | | 0.126 | | 0.012 |
| ebi-a-GCST004622 | finn-b-NAFLD | Reticulocyte count | Nonalcoholic fatty liver disease | rs482393 | | 0.315 | | 0.125 | | 0.012 |
| ebi-a-GCST004622 | finn-b-NAFLD | Reticulocyte count | Nonalcoholic fatty liver disease | rs378974 | | 0.316 | | 0.126 | | 0.012 |
| ebi-a-GCST004622 | finn-b-NAFLD | Reticulocyte count | Nonalcoholic fatty liver disease | rs8013143 | | 0.317 | | 0.126 | | 0.012 |
| ebi-a-GCST004622 | finn-b-NAFLD | Reticulocyte count | Nonalcoholic fatty liver disease | rs34592828 | | 0.316 | | 0.126 | | 0.012 |
| ebi-a-GCST004622 | finn-b-NAFLD | Reticulocyte count | Nonalcoholic fatty liver disease | rs6494407 | | 0.315 | | 0.126 | | 0.012 |
| ebi-a-GCST004622 | finn-b-NAFLD | Reticulocyte count | Nonalcoholic fatty liver disease | rs62466318 | | 0.315 | | 0.126 | | 0.012 |
| ebi-a-GCST004622 | finn-b-NAFLD | Reticulocyte count | Nonalcoholic fatty liver disease | rs12893668 | | 0.315 | | 0.126 | | 0.012 |
| ebi-a-GCST004622 | finn-b-NAFLD | Reticulocyte count | Nonalcoholic fatty liver disease | rs8017385 | | 0.315 | | 0.126 | | 0.012 |
| ebi-a-GCST004622 | finn-b-NAFLD | Reticulocyte count | Nonalcoholic fatty liver disease | rs732526 | | 0.314 | | 0.126 | | 0.012 |
| ebi-a-GCST004622 | finn-b-NAFLD | Reticulocyte count | Nonalcoholic fatty liver disease | rs3870341 | | 0.314 | | 0.126 | | 0.013 |
| ebi-a-GCST004622 | finn-b-NAFLD | Reticulocyte count | Nonalcoholic fatty liver disease | rs6914805 | | 0.314 | | 0.126 | | 0.013 |
| ebi-a-GCST004622 | finn-b-NAFLD | Reticulocyte count | Nonalcoholic fatty liver disease | rs4853525 | | 0.313 | | 0.126 | | 0.013 |
| ebi-a-GCST004622 | finn-b-NAFLD | Reticulocyte count | Nonalcoholic fatty liver disease | rs4554318 | | 0.313 | | 0.126 | | 0.013 |
| ebi-a-GCST004622 | finn-b-NAFLD | Reticulocyte count | Nonalcoholic fatty liver disease | rs11105328 | | 0.312 | | 0.126 | | 0.013 |
| ebi-a-GCST004622 | finn-b-NAFLD | Reticulocyte count | Nonalcoholic fatty liver disease | rs10067881 | | 0.314 | | 0.126 | | 0.013 |
| ebi-a-GCST004622 | finn-b-NAFLD | Reticulocyte count | Nonalcoholic fatty liver disease | rs4778581 | | 0.312 | | 0.126 | | 0.013 |
| ebi-a-GCST004622 | finn-b-NAFLD | Reticulocyte count | Nonalcoholic fatty liver disease | rs17272694 | | 0.312 | | 0.126 | | 0.013 |
| ebi-a-GCST004622 | finn-b-NAFLD | Reticulocyte count | Nonalcoholic fatty liver disease | rs2076085 | | 0.313 | | 0.126 | | 0.013 |
| ebi-a-GCST004622 | finn-b-NAFLD | Reticulocyte count | Nonalcoholic fatty liver disease | rs2737265 | | 0.312 | | 0.126 | | 0.013 |
| ebi-a-GCST004622 | finn-b-NAFLD | Reticulocyte count | Nonalcoholic fatty liver disease | rs2145943 | | 0.312 | | 0.126 | | 0.013 |
| ebi-a-GCST004622 | finn-b-NAFLD | Reticulocyte count | Nonalcoholic fatty liver disease | rs6997458 | | 0.311 | | 0.126 | | 0.013 |
| ebi-a-GCST004622 | finn-b-NAFLD | Reticulocyte count | Nonalcoholic fatty liver disease | rs11783469 | | 0.311 | | 0.126 | | 0.013 |
| ebi-a-GCST004622 | finn-b-NAFLD | Reticulocyte count | Nonalcoholic fatty liver disease | rs9920 | | 0.312 | | 0.126 | | 0.013 |
| ebi-a-GCST004622 | finn-b-NAFLD | Reticulocyte count | Nonalcoholic fatty liver disease | rs113731836 | | 0.311 | | 0.126 | | 0.013 |
| ebi-a-GCST004622 | finn-b-NAFLD | Reticulocyte count | Nonalcoholic fatty liver disease | rs10863511 | | 0.311 | | 0.126 | | 0.013 |
| ebi-a-GCST004622 | finn-b-NAFLD | Reticulocyte count | Nonalcoholic fatty liver disease | rs3817776 | | 0.312 | | 0.126 | | 0.013 |
| ebi-a-GCST004622 | finn-b-NAFLD | Reticulocyte count | Nonalcoholic fatty liver disease | rs9535495 | | 0.311 | | 0.126 | | 0.014 |
| ebi-a-GCST004622 | finn-b-NAFLD | Reticulocyte count | Nonalcoholic fatty liver disease | rs2857078 | | 0.312 | | 0.126 | | 0.014 |
| ebi-a-GCST004622 | finn-b-NAFLD | Reticulocyte count | Nonalcoholic fatty liver disease | rs2377585 | | 0.311 | | 0.126 | | 0.014 |
| ebi-a-GCST004622 | finn-b-NAFLD | Reticulocyte count | Nonalcoholic fatty liver disease | rs6889211 | | 0.310 | | 0.126 | | 0.014 |
| ebi-a-GCST004622 | finn-b-NAFLD | Reticulocyte count | Nonalcoholic fatty liver disease | rs111247693 | | 0.310 | | 0.126 | | 0.014 |
| ebi-a-GCST004622 | finn-b-NAFLD | Reticulocyte count | Nonalcoholic fatty liver disease | rs2247856 | | 0.310 | | 0.126 | | 0.014 |
| ebi-a-GCST004622 | finn-b-NAFLD | Reticulocyte count | Nonalcoholic fatty liver disease | rs174535 | | 0.309 | | 0.126 | | 0.014 |
| ebi-a-GCST004622 | finn-b-NAFLD | Reticulocyte count | Nonalcoholic fatty liver disease | rs140522 | | 0.309 | | 0.126 | | 0.014 |
| ebi-a-GCST004622 | finn-b-NAFLD | Reticulocyte count | Nonalcoholic fatty liver disease | rs1064833 | | 0.309 | | 0.126 | | 0.014 |
| ebi-a-GCST004622 | finn-b-NAFLD | Reticulocyte count | Nonalcoholic fatty liver disease | rs3087969 | | 0.310 | | 0.126 | | 0.014 |
| ebi-a-GCST004622 | finn-b-NAFLD | Reticulocyte count | Nonalcoholic fatty liver disease | rs73652622 | | 0.309 | | 0.126 | | 0.014 |
| ebi-a-GCST004622 | finn-b-NAFLD | Reticulocyte count | Nonalcoholic fatty liver disease | rs780142 | | 0.309 | | 0.126 | | 0.014 |
| ebi-a-GCST004622 | finn-b-NAFLD | Reticulocyte count | Nonalcoholic fatty liver disease | rs6474359 | | 0.309 | | 0.126 | | 0.014 |
| ebi-a-GCST004622 | finn-b-NAFLD | Reticulocyte count | Nonalcoholic fatty liver disease | rs77822621 | | 0.308 | | 0.126 | | 0.014 |
| ebi-a-GCST004622 | finn-b-NAFLD | Reticulocyte count | Nonalcoholic fatty liver disease | rs3765296 | | 0.308 | | 0.126 | | 0.014 |
| ebi-a-GCST004622 | finn-b-NAFLD | Reticulocyte count | Nonalcoholic fatty liver disease | rs113131010 | | 0.309 | | 0.126 | | 0.014 |
| ebi-a-GCST004622 | finn-b-NAFLD | Reticulocyte count | Nonalcoholic fatty liver disease | rs696281 | | 0.309 | | 0.126 | | 0.014 |
| ebi-a-GCST004622 | finn-b-NAFLD | Reticulocyte count | Nonalcoholic fatty liver disease | rs9739640 | | 0.308 | | 0.126 | | 0.015 |
| ebi-a-GCST004622 | finn-b-NAFLD | Reticulocyte count | Nonalcoholic fatty liver disease | rs753778 | | 0.308 | | 0.126 | | 0.015 |
| ebi-a-GCST004622 | finn-b-NAFLD | Reticulocyte count | Nonalcoholic fatty liver disease | rs2709802 | | 0.307 | | 0.126 | | 0.015 |
| ebi-a-GCST004622 | finn-b-NAFLD | Reticulocyte count | Nonalcoholic fatty liver disease | rs1613662 | | 0.307 | | 0.126 | | 0.015 |
| ebi-a-GCST004622 | finn-b-NAFLD | Reticulocyte count | Nonalcoholic fatty liver disease | rs75810210 | | 0.307 | | 0.126 | | 0.015 |
| ebi-a-GCST004622 | finn-b-NAFLD | Reticulocyte count | Nonalcoholic fatty liver disease | rs7896547 | | 0.309 | | 0.127 | | 0.015 |
| ebi-a-GCST004622 | finn-b-NAFLD | Reticulocyte count | Nonalcoholic fatty liver disease | rs55931012 | | 0.307 | | 0.126 | | 0.015 |
| ebi-a-GCST004622 | finn-b-NAFLD | Reticulocyte count | Nonalcoholic fatty liver disease | rs11048456 | | 0.307 | | 0.126 | | 0.015 |
| ebi-a-GCST004622 | finn-b-NAFLD | Reticulocyte count | Nonalcoholic fatty liver disease | rs1923366 | | 0.307 | | 0.126 | | 0.015 |
| ebi-a-GCST004622 | finn-b-NAFLD | Reticulocyte count | Nonalcoholic fatty liver disease | rs74180173 | | 0.308 | | 0.127 | | 0.015 |
| ebi-a-GCST004622 | finn-b-NAFLD | Reticulocyte count | Nonalcoholic fatty liver disease | rs73369896 | | 0.306 | | 0.126 | | 0.015 |
| ebi-a-GCST004622 | finn-b-NAFLD | Reticulocyte count | Nonalcoholic fatty liver disease | rs17476364 | | 0.309 | | 0.127 | | 0.015 |
| ebi-a-GCST004622 | finn-b-NAFLD | Reticulocyte count | Nonalcoholic fatty liver disease | rs11913573 | | 0.306 | | 0.126 | | 0.015 |
| ebi-a-GCST004622 | finn-b-NAFLD | Reticulocyte count | Nonalcoholic fatty liver disease | rs7908745 | | 0.306 | | 0.126 | | 0.015 |
| ebi-a-GCST004622 | finn-b-NAFLD | Reticulocyte count | Nonalcoholic fatty liver disease | rs72801474 | | 0.305 | | 0.126 | | 0.015 |
| ebi-a-GCST004622 | finn-b-NAFLD | Reticulocyte count | Nonalcoholic fatty liver disease | rs9260013 | | 0.306 | | 0.126 | | 0.015 |
| ebi-a-GCST004622 | finn-b-NAFLD | Reticulocyte count | Nonalcoholic fatty liver disease | rs34191159 | | 0.305 | | 0.126 | | 0.015 |
| ebi-a-GCST004622 | finn-b-NAFLD | Reticulocyte count | Nonalcoholic fatty liver disease | rs998584 | | 0.306 | | 0.126 | | 0.015 |
| ebi-a-GCST004622 | finn-b-NAFLD | Reticulocyte count | Nonalcoholic fatty liver disease | rs2361710 | | 0.306 | | 0.126 | | 0.015 |
| ebi-a-GCST004622 | finn-b-NAFLD | Reticulocyte count | Nonalcoholic fatty liver disease | rs10028187 | | 0.305 | | 0.126 | | 0.015 |
| ebi-a-GCST004622 | finn-b-NAFLD | Reticulocyte count | Nonalcoholic fatty liver disease | rs62573167 | | 0.305 | | 0.126 | | 0.015 |
| ebi-a-GCST004622 | finn-b-NAFLD | Reticulocyte count | Nonalcoholic fatty liver disease | rs12563740 | | 0.305 | | 0.126 | | 0.015 |
| ebi-a-GCST004622 | finn-b-NAFLD | Reticulocyte count | Nonalcoholic fatty liver disease | rs62197973 | | 0.304 | | 0.126 | | 0.015 |
| ebi-a-GCST004622 | finn-b-NAFLD | Reticulocyte count | Nonalcoholic fatty liver disease | rs68062772 | | 0.304 | | 0.126 | | 0.015 |
| ebi-a-GCST004622 | finn-b-NAFLD | Reticulocyte count | Nonalcoholic fatty liver disease | rs11545157 | | 0.305 | | 0.126 | | 0.016 |
| ebi-a-GCST004622 | finn-b-NAFLD | Reticulocyte count | Nonalcoholic fatty liver disease | rs7187994 | | 0.304 | | 0.126 | | 0.016 |
| ebi-a-GCST004622 | finn-b-NAFLD | Reticulocyte count | Nonalcoholic fatty liver disease | rs7692976 | | 0.304 | | 0.126 | | 0.016 |
| ebi-a-GCST004622 | finn-b-NAFLD | Reticulocyte count | Nonalcoholic fatty liver disease | rs3896594 | | 0.308 | | 0.128 | | 0.016 |
| ebi-a-GCST004622 | finn-b-NAFLD | Reticulocyte count | Nonalcoholic fatty liver disease | rs12454712 | | 0.304 | | 0.126 | | 0.016 |
| ebi-a-GCST004622 | finn-b-NAFLD | Reticulocyte count | Nonalcoholic fatty liver disease | rs753381 | | 0.303 | | 0.126 | | 0.016 |
| ebi-a-GCST004622 | finn-b-NAFLD | Reticulocyte count | Nonalcoholic fatty liver disease | rs74035509 | | 0.303 | | 0.126 | | 0.016 |
| ebi-a-GCST004622 | finn-b-NAFLD | Reticulocyte count | Nonalcoholic fatty liver disease | rs11678825 | | 0.303 | | 0.126 | | 0.016 |
| ebi-a-GCST004622 | finn-b-NAFLD | Reticulocyte count | Nonalcoholic fatty liver disease | rs10083830 | | 0.303 | | 0.126 | | 0.016 |
| ebi-a-GCST004622 | finn-b-NAFLD | Reticulocyte count | Nonalcoholic fatty liver disease | rs148311724 | | 0.303 | | 0.126 | | 0.016 |
| ebi-a-GCST004622 | finn-b-NAFLD | Reticulocyte count | Nonalcoholic fatty liver disease | rs9311474 | | 0.303 | | 0.126 | | 0.016 |
| ebi-a-GCST004622 | finn-b-NAFLD | Reticulocyte count | Nonalcoholic fatty liver disease | rs115421711 | | 0.306 | | 0.127 | | 0.016 |
| ebi-a-GCST004622 | finn-b-NAFLD | Reticulocyte count | Nonalcoholic fatty liver disease | rs73166632 | | 0.304 | | 0.126 | | 0.016 |
| ebi-a-GCST004622 | finn-b-NAFLD | Reticulocyte count | Nonalcoholic fatty liver disease | rs11647753 | | 0.302 | | 0.125 | | 0.016 |
| ebi-a-GCST004622 | finn-b-NAFLD | Reticulocyte count | Nonalcoholic fatty liver disease | rs1175550 | | 0.305 | | 0.127 | | 0.016 |
| ebi-a-GCST004622 | finn-b-NAFLD | Reticulocyte count | Nonalcoholic fatty liver disease | rs4606752 | | 0.301 | | 0.125 | | 0.016 |
| ebi-a-GCST004622 | finn-b-NAFLD | Reticulocyte count | Nonalcoholic fatty liver disease | rs35578928 | | 0.302 | | 0.125 | | 0.016 |
| ebi-a-GCST004622 | finn-b-NAFLD | Reticulocyte count | Nonalcoholic fatty liver disease | rs7432379 | | 0.302 | | 0.126 | | 0.016 |
| ebi-a-GCST004622 | finn-b-NAFLD | Reticulocyte count | Nonalcoholic fatty liver disease | rs68021656 | | 0.301 | | 0.125 | | 0.016 |
| ebi-a-GCST004622 | finn-b-NAFLD | Reticulocyte count | Nonalcoholic fatty liver disease | rs754205 | | 0.301 | | 0.125 | | 0.016 |
| ebi-a-GCST004622 | finn-b-NAFLD | Reticulocyte count | Nonalcoholic fatty liver disease | rs2562175 | | 0.298 | | 0.124 | | 0.016 |
| ebi-a-GCST004622 | finn-b-NAFLD | Reticulocyte count | Nonalcoholic fatty liver disease | rs79220007 | | 0.304 | | 0.126 | | 0.016 |
| ebi-a-GCST004622 | finn-b-NAFLD | Reticulocyte count | Nonalcoholic fatty liver disease | rs3184504 | | 0.306 | | 0.128 | | 0.016 |
| ebi-a-GCST004622 | finn-b-NAFLD | Reticulocyte count | Nonalcoholic fatty liver disease | rs17513363 | | 0.301 | | 0.126 | | 0.017 |
| ebi-a-GCST004622 | finn-b-NAFLD | Reticulocyte count | Nonalcoholic fatty liver disease | rs4806103 | | 0.300 | | 0.125 | | 0.017 |
| ebi-a-GCST004622 | finn-b-NAFLD | Reticulocyte count | Nonalcoholic fatty liver disease | rs10649609 | | 0.300 | | 0.126 | | 0.017 |
| ebi-a-GCST004622 | finn-b-NAFLD | Reticulocyte count | Nonalcoholic fatty liver disease | rs198325 | | 0.296 | | 0.124 | | 0.017 |
| ebi-a-GCST004622 | finn-b-NAFLD | Reticulocyte count | Nonalcoholic fatty liver disease | rs116113631 | | 0.302 | | 0.127 | | 0.017 |
| ebi-a-GCST004622 | finn-b-NAFLD | Reticulocyte count | Nonalcoholic fatty liver disease | rs4810067 | | 0.300 | | 0.126 | | 0.017 |
| ebi-a-GCST004622 | finn-b-NAFLD | Reticulocyte count | Nonalcoholic fatty liver disease | rs12981369 | | 0.293 | | 0.123 | | 0.017 |
| ebi-a-GCST004622 | finn-b-NAFLD | Reticulocyte count | Nonalcoholic fatty liver disease | rs146472986 | | 0.296 | | 0.124 | | 0.017 |
| ebi-a-GCST004622 | finn-b-NAFLD | Reticulocyte count | Nonalcoholic fatty liver disease | rs2745349 | | 0.296 | | 0.124 | | 0.017 |
| ebi-a-GCST004622 | finn-b-NAFLD | Reticulocyte count | Nonalcoholic fatty liver disease | rs875741 | | 0.301 | | 0.126 | | 0.017 |
| ebi-a-GCST004622 | finn-b-NAFLD | Reticulocyte count | Nonalcoholic fatty liver disease | rs11587735 | | 0.299 | | 0.126 | | 0.018 |
| ebi-a-GCST004622 | finn-b-NAFLD | Reticulocyte count | Nonalcoholic fatty liver disease | rs2280742 | | 0.298 | | 0.126 | | 0.018 |
| ebi-a-GCST004622 | finn-b-NAFLD | Reticulocyte count | Nonalcoholic fatty liver disease | rs72996113 | | 0.297 | | 0.125 | | 0.018 |
| ebi-a-GCST004622 | finn-b-NAFLD | Reticulocyte count | Nonalcoholic fatty liver disease | rs75759008 | | 0.296 | | 0.125 | | 0.018 |
| ebi-a-GCST004622 | finn-b-NAFLD | Reticulocyte count | Nonalcoholic fatty liver disease | rs1260326 | | 0.294 | | 0.125 | | 0.019 |
| ebi-a-GCST004622 | finn-b-NAFLD | Reticulocyte count | Nonalcoholic fatty liver disease | rs13101482 | | 0.297 | | 0.126 | | 0.019 |
| ebi-a-GCST004622 | finn-b-NAFLD | Reticulocyte count | Nonalcoholic fatty liver disease | rs114309882 | | 0.295 | | 0.126 | | 0.019 |
| ebi-a-GCST004622 | finn-b-NAFLD | Reticulocyte count | Nonalcoholic fatty liver disease | rs11014296 | | 0.303 | | 0.130 | | 0.019 |
| ebi-a-GCST004622 | finn-b-NAFLD | Reticulocyte count | Nonalcoholic fatty liver disease | rs9349205 | | 0.295 | | 0.126 | | 0.019 |
| ebi-a-GCST004622 | finn-b-NAFLD | Reticulocyte count | Nonalcoholic fatty liver disease | rs9272058 | | 0.292 | | 0.126 | | 0.020 |
| ebi-a-GCST004622 | finn-b-NAFLD | Reticulocyte count | Nonalcoholic fatty liver disease | rs112875651 | | 0.268 | | 0.117 | | 0.022 |
| ebi-a-GCST004622 | finn-b-NAFLD | Reticulocyte count | Nonalcoholic fatty liver disease | rs592423 | | 0.275 | | 0.128 | | 0.032 |
| ebi-a-GCST004622 | finn-b-NAFLD | Reticulocyte count | Nonalcoholic fatty liver disease | All | | 0.308 | | 0.125 | | 0.014 |
