# Supplementary material for: Blood cell parameters and risk of nonalcoholic fatty liver disease: a comprehensive Mendelian randomization study
Source: BMC Med Genomics. 2024 Apr 23;17:102. doi: 10.1186/s12920-024-01879-7 (PMC11040836; doi:10.1186/s12920-024-01879-7)
Supplement: Supplementary file 3 — Supplementary Material 3 [file 12920_2024_1879_MOESM3_ESM.doc]

| **Table S3 Estimation of causal effect of blood cells and circulating cytokines and growth factors** | | | | | | | | |
| --- | --- | --- | --- | --- | --- | --- | --- | --- |
| **id.exposure** | **id.outcome** | **Exposure** | **Outcome** | **Method** | **No.SNP** | **Beta** | **ES** | **p** |
| ebi-a-GCST004622 | ebi-a-GCST004420 | Reticulocyte count | CTACK levels | Inverse variance weighted | 135 | -0.054 | 0.058 | 0.346 |
| ebi-a-GCST004622 | ebi-a-GCST004421 | Reticulocyte count | beta-nerve growth factor levels | Inverse variance weighted | 135 | -0.034 | 0.058 | 0.562 |
| ebi-a-GCST004622 | ebi-a-GCST004422 | Reticulocyte count | Vascular endothelial growth factor levels | Inverse variance weighted | 135 | 0.021 | 0.045 | 0.641 |
| ebi-a-GCST004622 | ebi-a-GCST004423 | Reticulocyte count | Macrophage Migration Inhibitory Factor levels | Inverse variance weighted | 134 | 0.025 | 0.058 | 0.663 |
| ebi-a-GCST004622 | ebi-a-GCST004424 | Reticulocyte count | TRAIL levels | Inverse variance weighted | 130 | 0.019 | 0.044 | 0.659 |
| ebi-a-GCST004622 | ebi-a-GCST004425 | Reticulocyte count | Tumor necrosis factor beta levels | Inverse variance weighted | 116 | -0.084 | 0.093 | 0.361 |
| ebi-a-GCST004622 | ebi-a-GCST004426 | Reticulocyte count | Tumor necrosis factor alpha levels | Inverse variance weighted | 135 | 0.039 | 0.061 | 0.527 |
| ebi-a-GCST004622 | ebi-a-GCST004427 | Reticulocyte count | Stromal-cell-derived factor 1 alpha levels | Inverse variance weighted | 135 | -0.004 | 0.041 | 0.930 |
| **ebi-a-GCST004622** | **ebi-a-GCST004428** | **Reticulocyte count** | **Stem cell growth factor beta levels** | **Inverse variance weighted** | **135** | **0.145** | **0.057** | **0.011** |
| ebi-a-GCST004622 | ebi-a-GCST004429 | Reticulocyte count | Stem cell factor levels | Inverse variance weighted | 135 | 0.030 | 0.042 | 0.484 |
| ebi-a-GCST004622 | ebi-a-GCST004430 | Reticulocyte count | Interleukin-16 levels | Inverse variance weighted | 135 | 0.058 | 0.058 | 0.315 |
| ebi-a-GCST004622 | ebi-a-GCST004431 | Reticulocyte count | RANTES levels | Inverse variance weighted | 135 | -0.037 | 0.063 | 0.555 |
| ebi-a-GCST004622 | ebi-a-GCST004432 | Reticulocyte count | Platelet-derived growth factor BB levels | Inverse variance weighted | 135 | 0.057 | 0.041 | 0.157 |
| ebi-a-GCST004622 | ebi-a-GCST004433 | Reticulocyte count | Macrophage inflammatory protein 1b levels | Inverse variance weighted | 134 | -0.018 | 0.047 | 0.696 |
| ebi-a-GCST004622 | ebi-a-GCST004434 | Reticulocyte count | Macrophage inflammatory protein 1a levels | Inverse variance weighted | 135 | -0.060 | 0.061 | 0.329 |
| ebi-a-GCST004622 | ebi-a-GCST004435 | Reticulocyte count | Monokine induced by gamma interferon levels | Inverse variance weighted | 134 | 0.016 | 0.057 | 0.775 |
| ebi-a-GCST004622 | ebi-a-GCST004436 | Reticulocyte count | Macrophage colony stimulating factor levels | Inverse variance weighted | 134 | -0.101 | 0.070 | 0.149 |
| ebi-a-GCST004622 | ebi-a-GCST004437 | Reticulocyte count | Monocyte chemoattractant protein-3 levels | Inverse variance weighted | 128 | 0.131 | 0.107 | 0.217 |
| ebi-a-GCST004622 | ebi-a-GCST004438 | Reticulocyte count | Monocyte chemoattractant protein-1 levels | Inverse variance weighted | 135 | -0.001 | 0.040 | 0.978 |
| ebi-a-GCST004622 | ebi-a-GCST004439 | Reticulocyte count | Interleukin-12p70 levels | Inverse variance weighted | 135 | -0.004 | 0.043 | 0.919 |
| ebi-a-GCST004622 | ebi-a-GCST004440 | Reticulocyte count | Interferon gamma-induced protein 10 levels | Inverse variance weighted | 135 | 0.023 | 0.060 | 0.700 |
| ebi-a-GCST004622 | ebi-a-GCST004441 | Reticulocyte count | Interleukin-18 levels | Inverse variance weighted | 135 | 0.043 | 0.058 | 0.458 |
| ebi-a-GCST004622 | ebi-a-GCST004442 | Reticulocyte count | Interleukin-17 levels | Inverse variance weighted | 135 | 0.001 | 0.042 | 0.987 |
| ebi-a-GCST004622 | ebi-a-GCST004443 | Reticulocyte count | Interleukin-13 levels | Inverse variance weighted | 135 | 0.011 | 0.058 | 0.850 |
| ebi-a-GCST004622 | ebi-a-GCST004444 | Reticulocyte count | Interleukin-10 levels | Inverse variance weighted | 135 | 0.027 | 0.042 | 0.524 |
| ebi-a-GCST004622 | ebi-a-GCST004445 | Reticulocyte count | Interleukin-8 levels | Inverse variance weighted | 134 | 0.073 | 0.058 | 0.210 |
| ebi-a-GCST004622 | ebi-a-GCST004446 | Reticulocyte count | Interleukin-6 levels | Inverse variance weighted | 135 | 0.021 | 0.039 | 0.591 |
| ebi-a-GCST004622 | ebi-a-GCST004447 | Reticulocyte count | Interleukin-1-receptor antagonist levels | Inverse variance weighted | 134 | 0.025 | 0.058 | 0.662 |
| ebi-a-GCST004622 | ebi-a-GCST004448 | Reticulocyte count | Interleukin-1-beta levels | Inverse variance weighted | 135 | -0.016 | 0.048 | 0.741 |
| ebi-a-GCST004622 | ebi-a-GCST004449 | Reticulocyte count | Hepatocyte growth factor levels | Inverse variance weighted | 135 | 0.052 | 0.042 | 0.215 |
| ebi-a-GCST004622 | ebi-a-GCST004450 | Reticulocyte count | Interleukin-9 levels | Inverse variance weighted | 135 | 0.029 | 0.064 | 0.655 |
| ebi-a-GCST004622 | ebi-a-GCST004451 | Reticulocyte count | Interleukin-7 levels | Inverse variance weighted | 135 | 0.016 | 0.062 | 0.792 |
| ebi-a-GCST004622 | ebi-a-GCST004452 | Reticulocyte count | Interleukin-5 levels | Inverse variance weighted | 135 | 0.011 | 0.061 | 0.857 |
| ebi-a-GCST004622 | ebi-a-GCST004453 | Reticulocyte count | Interleukin-4 levels | Inverse variance weighted | 135 | 0.016 | 0.041 | 0.702 |
| ebi-a-GCST004622 | ebi-a-GCST004454 | Reticulocyte count | Interleukin-2 receptor antagonist levels | Inverse variance weighted | 135 | 0.034 | 0.057 | 0.557 |
| ebi-a-GCST004622 | ebi-a-GCST004455 | Reticulocyte count | Interleukin-2 levels | Inverse variance weighted | 135 | 0.001 | 0.058 | 0.984 |
| ebi-a-GCST004622 | ebi-a-GCST004456 | Reticulocyte count | Interferon gamma levels | Inverse variance weighted | 135 | -0.034 | 0.044 | 0.437 |
| ebi-a-GCST004622 | ebi-a-GCST004457 | Reticulocyte count | Growth-regulated protein alpha levels | Inverse variance weighted | 135 | 0.018 | 0.058 | 0.762 |
| ebi-a-GCST004622 | ebi-a-GCST004458 | Reticulocyte count | Granulocyte-colony stimulating factor levels | Inverse variance weighted | 134 | 0.026 | 0.040 | 0.512 |
| ebi-a-GCST004622 | ebi-a-GCST004459 | Reticulocyte count | Fibroblast growth factor basic levels | Inverse variance weighted | 134 | 0.020 | 0.045 | 0.666 |
| ebi-a-GCST004622 | ebi-a-GCST004460 | Reticulocyte count | Eotaxin levels | Inverse variance weighted | 135 | 0.002 | 0.040 | 0.955 |
| **Multivariate MR** | | | | | | | | |
| **id.exposure** | **id.outcome** | **Exposure** | **Outcome** | **No.SNP** | **Beta** | **SE** | **p** | |
| ebi-a-GCST004428 | finn-b-NAFLD | Stem cell growth factor beta levels | Nonalcoholic fatty liver disease | 3 | 0.131 | 0.119 | 0.273 | |
| ebi-a-GCST004622 | finn-b-NAFLD | Reticulocyte count | Nonalcoholic fatty liver disease | 136 | 0.270 | 0.117 | 0.021 | |
| **Intermediary MR Analysis** | | | | | | | | |
| **Exprosure (E)** | | **Mediator (M)** | | **Mediation effect (OR/95% CI)** | | **p** | | |
| Reticulocyte count | | Stem cell growth factor beta levels | | 1.040  (0.994，1.087) | | 0.087 | | |
